# Clinical and Laboratory Profile of Enteric Fever in Children From a Tertiary Care Centre in Odisha, Eastern India

**DOI:** 10.7759/cureus.12826

**Published:** 2021-01-20

**Authors:** Jyoti Ranjan Behera, Amit R Rup, Arun K Dash, Sanjay Kumar Sahu, Abhinav Gaurav, Abhas Gupta

**Affiliations:** 1 Pediatrics, Kalinga Institute of Industrial Technology, Bhubaneswar, IND; 2 Pediatrics, Kalinga Institute of Medical Sciences, Bhubaneswar, IND

**Keywords:** enteric fever, salmonella, eosinopenia, narst

## Abstract

Background: Enteric fever is a major health problem in developing countries. Varied clinical presentation leads to diagnostic dilemmas resulting in fatal complications.

Objective: To determine the socio-demographic, clinical manifestations, complications, antibiotic sensitivity pattern, treatment, and outcome in hospitalized enteric fever patients.

Methods: A retrospective case record analysis of hospitalized patients in the age group one to 14 years with a discharge diagnosis of enteric fever was done in a tertiary care centre of Odisha over a period of three years (January 2017 to December 2019).

Results: Of 112 patients, 75% of children belonged to the six to 14 years age group with a mean age of 7.6 +/- 3.6 years and a male to female ratio of 1.66:1. The peak of cases was seen during the month of January to June with 94% of cases occurring in low and middle socioeconomic status. The commonest presentation was fever in 98.21%; other features were vomiting (39.29%), pain in abdomen (21.43%), diarrhoea (26.79%), and anorexia (14.29%). Eosinopenia was found in 58.93%, transaminitis in 30.36%, and raised CRP in 73.21%. In 30 children blood culture was positive with sensitivity to third-generation cephalosporin. All isolates were nalidixic acid-resistant *Salmonella* Typhi (NARST). Complications were seen in 21.42%. All recovered and two left against medical advice.

Conclusion: Enteric fever is a major threat in the paediatric age group. Early clinical diagnosis with rational use of antibiotics according to sensitivity pattern is important. Improved hygiene, vaccination, and awareness among people is necessary for prevention.

## Introduction

Enteric fever is a multi-systemic tropical infectious disease. Causative organisms are *Salmonella enterica* serotype Typhi (S. typhi) or *Salmonella enterica* serotype Paratyphi A, B, or C. It is prevalent in most underdeveloped countries, with India having a high disease burden of 214.2 per 100,000 individuals per year [1]. Endemicity in developing countries is attributed to the low standard of living, poor hygiene practices, poor sanitation, contaminated water sources, and lack of universal vaccination. In children, the common age group affected is between five to 19 years, but in some endemic areas of Asia, it is also common in children less than two years [2]. Clinical manifestations are non-specific, which may delay the diagnosis and treatment leading to fatal complications. Presenting complaints vary from mild constitutional symptoms to severe complications involving multiple organs. Clinical suspicion is pivotal for diagnosis. Common presentations are fever, vomiting, diarrhoea, abdominal pain, cough, headache, and lethargy. The gold standard for diagnosis is blood culture, but in 70% the culture is negative due to injudicious use of antibiotics before admission [3]. This study intended to determine the socio-demographic factors, clinical profile, complications, sensitivity pattern of antibiotics, the treatment used, and outcome in hospitalized children with enteric fever.

## Materials and methods

This study was a retrospective analysis, done in a tertiary care centre in Odisha, eastern India, over a period of three years (January 2017 to December 2019). Case records of children between one and 14 years having discharge diagnosis of enteric fever were analyzed. The inclusion criteria were the presence of signs and symptoms suggestive of enteric fever and blood culture positive for *Salmonella* or positive rapid diagnostic test (Typhidot IgM, sensitivity 84% and specificity 79%) or Widal positive (TO titer >1:160 or TH titer >1:160) [4]. Information on the socio-demographic profile, immunisation status, presenting complaints, duration of illness, length of hospital stay, clinical profile, laboratory data, treatment given, complication, and outcome were extracted from hospital records and collated on a Microsoft Excel sheet. Analysis of the collected data was done using Stata version 15.1 (StataCorp., College Station, TX, USA).

The variable definitions used in this study were: leucopenia: total leucocyte count <4000 cells/mm3; leucocytosis: total leucocyte count >11,000 cells/mm3; eosinophilia: absolute eosinophil count (AEC) >450 cells/mm3; eosinopenia: absence of eosinophils in the peripheral smear; transaminitis: raised liver enzymes >2 times of the normal; hyponatremia: serum sodium <135 mEq/L; hypokalemia: serum potassium <3.5 mEq/L; raised C-reactive protein (CRP): CRP level >6 mg/L; relapse: recurrence of fever within two weeks after stoppage of antibiotics.

## Results

**Figure 1:**
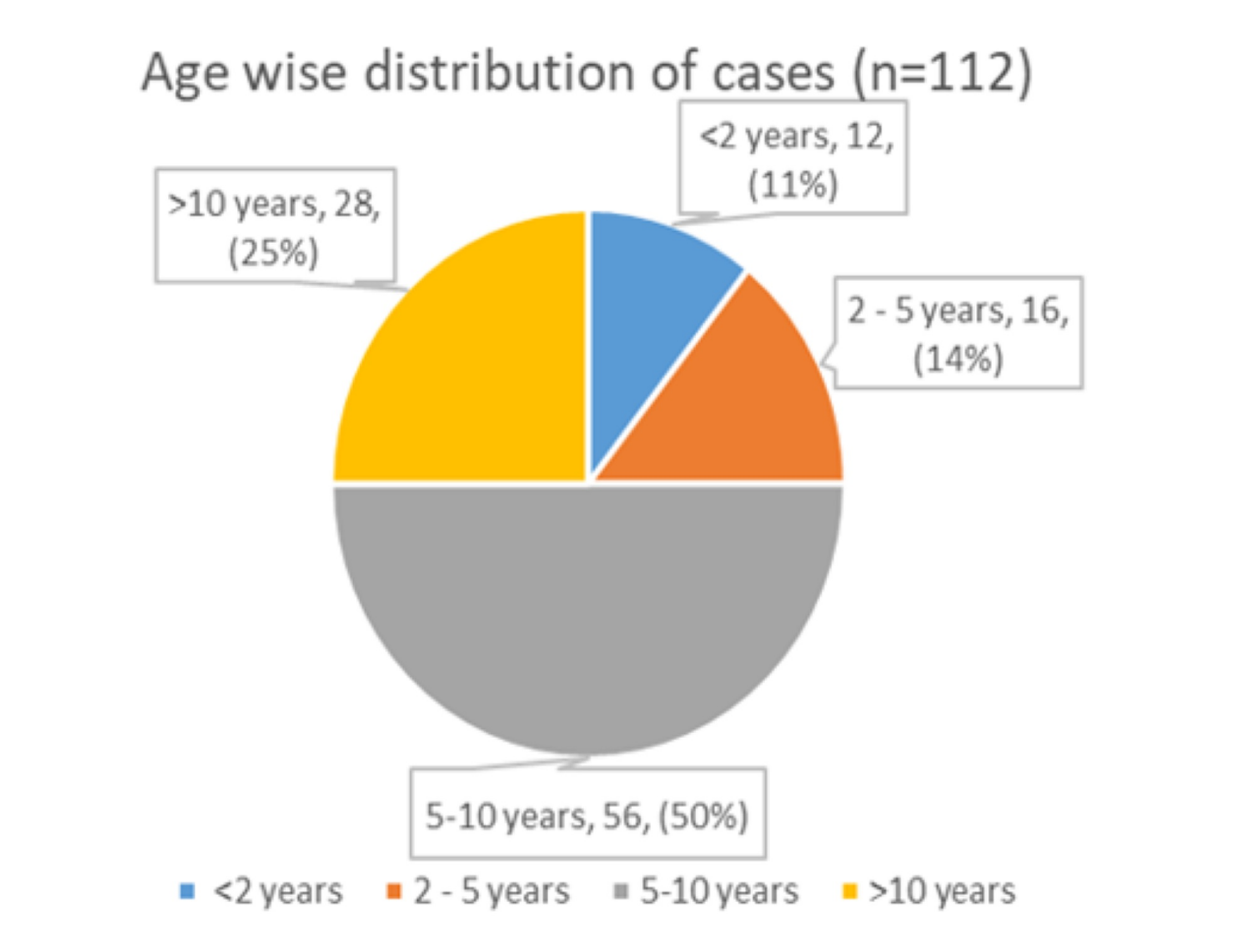
Age wise distribution of cases

**Figure 2:**
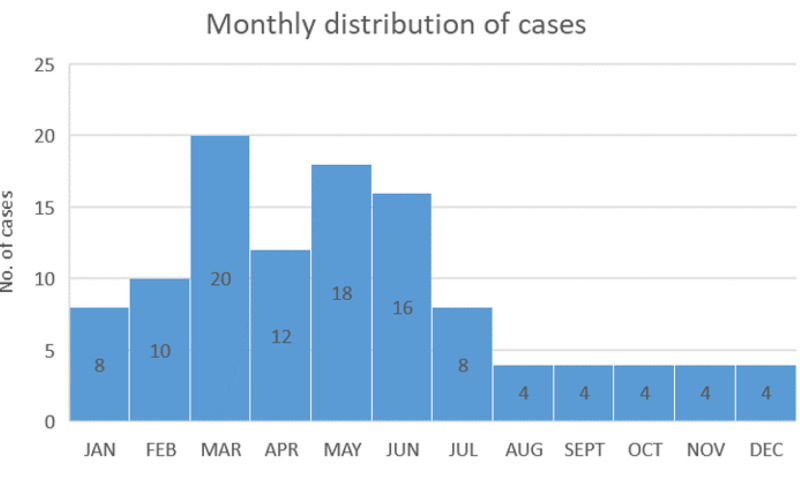
Month wise distribution of cases

Case records of children discharged with a diagnosis of enteric fever were analysed and 112 children in the age range of one to 14 years were included in the study. The mean age of presentation was 7.6 +/- 3.6 years. Seventy-five percent of the children belong to the six to 14 years age group. Age-wise distribution is given in Figure 1. Boys and girls constituted 62.5% and 37.5%, respectively, with a male to female ratio of 1.66:1. Middle socio-economic status (SES) constituted 71.43% whereas 23.21% were from the low and 5.36% were from the higher group. Seasonal variation was observed with the peak during the months of January to June (Figure 2).

Eleven children (9.8%) had prior history of Typhoid vaccination. Out of all children, 110 (98.21%) presented with fever for more than five days duration. Other presenting features were vomiting (44; 39.29%), pain abdomen (24; 21.43%), diarrhoea (30; 26.79%) anorexia (16; 14.29%), cough (22; 19.64%) and headache (eight; 7.14%). Most of the children had received oral antibiotics before hospitalization. On admission, necessary investigations including blood culture were sent before the initiation of antibiotics. Major clinical findings were pallor in 29 (25.8%), icterus in 12 (10.71%), coated tongue in two (1.79%), hepatomegaly in 18 (16.07%), and splenomegaly in 10 (8.93%) children. Investigations revealed anemia in 48 (42.86%), leucopenia in 12 (10.71%) leucocytosis in 22 (19.64%), thrombocytopenia in 16 (14.29%), pancytopenia in eight (7.14%), eosinopenia in 66 (58.93%), transaminitis in 34 (30.36%), hyponatremia in 14 (12.50%), hypokalemia in 10 (8.93%), and raised CRP in 82 (73.21%) children. Signs, symptoms, investigations, and complications are depicted in Table 1.

**Table 1:** Clinical features, laboratory findings and complications

Symptoms	n	%
Fever	110	(98.21%)
Vomiting	44	(39.29%)
Pain abdomen	24	(21.43%)
Diarrhoea	30	(26.79%)
Anorexia	16	(14.29%)
Cough	22	(19.64%)
Headache	8	(7.14%)
Signs		
Pallor	29	25.8
Icterus	12	10.71
Coated tongue	2	1.79
Hepatomegaly	18	16.07
Splenomegaly	10	8.93
Investigations		
Anemia	48	42.86
Leucopenia	12	10.71
Leucocytosis	22	19.64
Thrombocytopenia	16	14.29
Pancytopenia	8	7.14
Eosinopenia	66	58.93
Transaminitis	34	30.36
Hyponatremia	14	12.50
Hypokalemia	10	8.93
Raised c-reactive protein (CRP)	82	73.21
Complications		
Bronchitis	8	7.14
Bronchopneumonia	2	1.79
Encephalopathy	2	1.79
Hepatitis	12	10.71

Widal test was positive in 32 children, Typhidot was positive in 64, and blood culture in 30. Blood culture and Typhidot were positive in six, blood culture and Widal in six, and Widal and Typhidot in two but no children had all three positive. All strains isolated were sensitive to ceftriaxone, cotrimoxazole, meropenem, and piperacillin + tazobactam. Nalidixic acid-resistant *Salmonella* Typhi (NARST) was observed in all culture positive cases. The antibiotic sensitivity pattern is depicted in Table 2.

**Table 2:** Antibiotic sensitivity pattern

Drug	Sensitive	Resistance
Ceftriaxone	30	0
Ciprofloxacin	6	24
Levofloxacin	10	20
Nalidixic acid	0	30
Co-trimoxazole	30	0
Piperacillin + Tazobactum	30	0
Meropenem	30	0

Most of the children (94.6%) were started on intravenous ceftriaxone along with other supportive measures. After defervescence and improved oral intake of the child, it was switched over to oral cefixime. Treatment was continued for a total duration of 14 days. Children who had no improvement after five days of therapy were added oral azithromycin. Meropenem was started in four (3.57%) children and piperacillin + tazobactam in two (1.79%) on the basis of severity of illness during admission. The mean fever defervescence period was 3.5 +/- 2.09 days. Complications were seen in 24 (21.42%) children. Bronchitis was seen in eight (7.14%), bronchopneumonia in two (1.79%), encephalopathy in two (1.79%) and hepatitis in 12 (10.71%) children. The average duration of hospitalization was seven days (interquartile range [IQR] 6-9). Out of 112 children, all recovered and two left against medical advice.

## Discussion

In developing countries, enteric fever causes significant morbidity in children. This study aims to identify various socio-demographic, clinical, and laboratory parameters of typhoid which are significant. Male predominance found in this study is very similar to the finding of Rabasa et al. and Ramaswamy et al. [3,5]. This may be due to overindulgence in outdoor activities in more boys than girls, therefore exposure to sources of infection. Most of the cases were detected during the months of January to June in contrast to earlier observations by Siddiqui et al. (study from Karachi, Pakistan) where the peak was reported in October, which can be attributed to environmental and geographical factors [6]. The majority of children were between five and 10 years of age similar to a study done by Walia et al. [7]. In the under two year age group, the incidence of 10.7% highlights the importance of early immunization required for prevention of the disease and also supports the findings by Monorama et al. and Saha et al. [8,9]. Another study from Dhaka, Bangladesh by Hyder et al. was done exclusively on children under two years. They detected 40 cases in a span of 10 months despite popular belief that enteric fever is a rarity in this age group [10]. Socioeconomic status has a very important role to play in hygiene and sanitation and hence tends to affect the incidence of enteric fever. This was reflected in this study as 94% of the affected children belonged to lower or middle socio-economic status, which resembles the findings of a Nigerian study [3].

Fever was the commonest symptom seen in almost all (98.21%) cases and gastrointestinal symptoms like vomiting, diarrhoea, and pain in abdomen were the other notable clinical features, similar to observations made by other researchers [5-7,11]. Total leukocyte count was normal in 70% of our cases which is in concordance with the Indian Academy of Paediatrics guidelines for the diagnosis of enteric fever [12]. Eosinopenia was detected in 58% of cases, an even higher incidence (72%) was observed in the findings of Ramaswamy et al. [5]. Hence eosinopenia can be used as a marker for diagnosis. CRP, an acute phase reactant, was elevated in around three-quarters of patients, which was not commonly found in other studies. Observed transaminitis was much less than in Chitkara et al. (30% vs. 77%) [13]. Blood culture, which is considered as the gold standard for diagnosis, was positive in 26.7% which was also observed by many other Indian studies [5,14]. But a recently published Delhi-based study revealed very high (61%) culture positivity which can be attributed to various factors like quick access to a medical facility, lesser antibiotic use before, and probably good culture technique [13]. The sensitivity pattern was similar to the above-described study as all were fully sensitive to third-generation cephalosporin. However, NARST percentages were a striking contrast (100% in this study to 51.9% in Chitkara et al.) which warrants judicial and restricted use of quinolones [13].

For management of enteric fever, the Indian Academy of Pediatrics (IAP) Task Force report recommends the usage of parenteral ceftriaxone or oral cefixime for 14 days as first-line treatment [15]. In this study, 70% of children were successfully treated with this regimen with 24% requiring additional azithromycin. The mean duration of defervescence and hospital stay were comparable with previous studies [5]. Complications were recorded in 21% of cases with hepatitis being the commonest with 10.71% whereas only 1.3% had complications in the Delhi-based study [13]. A high rate of complications in this study can be attributed to delayed presentation to the hospital, however there was no mortality or relapse.

This study signifies that eosinopenia and elevated CRP can be used as supplementary diagnostic markers along with Widal and Typhidot test in culture-negative patients having clinical features compatible with enteric fever. A retrospective study with a small sample size not representing the target population was the major drawback of this study. 

## Conclusions

Enteric fever is still a big threat to the paediatric population with many requiring admission in tropical countries like India. A high index of clinical suspicion is crucial for diagnosis as none of the diagnostic modalities are sensitive enough. Proper use of antibiotics and keeping an eye on local sensitivity patterns is the key to therapeutic success. Public health interventions like clean drinking water supply, maintenance of hygiene, regular handwashing, and vaccination at an early age shall help for the prevention of enteric fever in children.

## References

[1] Ochiai RL, Acosta CJ, Danovaro-Holliday M (2008). A study of typhoid fever in five Asian countries: disease burden and implications for controls. Bull World Health Organ.

[2] Laishram N, Singh PA (2016). Clinical profile of enteric fever in children. J Evolution Med Dent Sci.

[3] Rabasa A, Mava Y, Pius S, Timothy SY, Baba UA (2013). Typhoid fever in children: clinical presentation and risk factors. Niger J Paediatr.

[4] Wijedoru L, Mallett S, Parry CM (2017). Rapid diagnostic tests for typhoid and paratyphoid (enteric) fever. Cochrane Database Syst Rev.

[5] Ganesh R, Janakiraman L, Vasanthi T, Sathiyasekeran M (2010). Profile of typhoid fever in children from a tertiary care hospital in Chennai-South India. Indian J Pediatr.

[6] Siddiqui FJ, Rabbani F, Hasan R, Nizami SQ, Bhutta ZA (2006). Typhoid fever in children: some epidemiological considerations from Karachi, Pakistan. Int J Infect Dis.

[7] Walia M, Gaind R, Mehta R, Paul P, Aggarwal P, Kalaivani M (2005). Current perspectives of enteric fever:a hospitl based study from India. Ann Trop Paediatr.

[8] Verma M, Chhatwal J, Saini V, Singh T (1996). Enteric fever below 2 years of age. Indian Pediatr.

[9] Saha SK, Baqui AH, Hanif M (2001). Typhoid fever in Bangladesh: implications for vaccination policy. Pediatr Infect Dis J.

[10] Hyder RT, Yasmeen BN, Ahmed S, Islam MS, Islam F (2013). Clinical profile and outcome of enteric fever in hospitalized children aged 6 months to 2 years. North Int Med Coll J.

[11] Sinha A, Sazawal S, Kumar R (1999). Typhoid fever in children aged less than 5 years. Lancet.

[12] IAP Task Force (2006). IAP Task Force report: diagnosis of enteric fever in children. Indian Pediatr.

[13] Chitkara AJ, Chitkara S, Narang PS, Sundharam M, Goyal M (2019). Clinico-bacteriological profile of typhoid fever in a private sector hospital in New Delhi. Indian Pediatr.

[14] Ray P, Sharma J, Marak RK, Garg RK (2006). Predictive efficacy of nalidixic acid resistance as a marker of fluoroquinolone resistance in Salmonella enterica var Typhi. Indian J Med Res.

[15] IAP Task Force (2006). IAP Task Force report: management of enteric fever in children. Indian Pediatr.

